# The proposed role of plasma NT pro-brain natriuretic peptide in assessing cardiac remodelling in hypertensive African subjects

**DOI:** 10.5830/CVJA-2014-050

**Published:** 2014

**Authors:** Dike B Ojji, Lionel H Opie, Sandrine Lecour, Lydia Lacerda, Karen Sliwa, Olusoji M Adeyemi

**Affiliations:** Cardiology Unit, Department of Medicine, University of Abuja Teaching Hospital, Gwagwalada, Abuja; Hatter Institute for Cardiovascular Research in Africa, MRC Inter-Cape Heart Unit, Department of Medicine, Faculty of Health Sciences, University of Cape Town, South Africa; Hatter Institute for Cardiovascular Research in Africa, MRC Inter-Cape Heart Unit, Department of Medicine, Faculty of Health Sciences, University of Cape Town, South Africa; Hatter Institute for Cardiovascular Research in Africa, MRC Inter-Cape Heart Unit, Department of Medicine, Faculty of Health Sciences, University of Cape Town, South Africa; Hatter Institute for Cardiovascular Research in Africa, MRC Inter-Cape Heart Unit, Department of Medicine, Faculty of Health Sciences, University of Cape Town, South Africa; Hatter Institute for Cardiovascular Research in Africa, MRC Inter-Cape Heart Unit, Department of Medicine, Faculty of Health Sciences, University of Cape Town, South Africa; Soweto Cardiovascular Research Unit, Faculty of Health Sciences, University of the Witwatersrand; Department of Medical Laboratory Sciences, University of Abuja Teaching Hospital, Gwagwalada, Abuja

**Keywords:** hypertension, cardiac remodeling, left ventricle, right ventricle, NT-proBNP

## Abstract

**Aim:**

Although plasma NT-proBNP differentiates hypertension (HT) with or without left ventricular hypertrophy (LVH) from hypertensive heart failure (HHF), most of the published data are based on studies in Western populations. Also, most previous studies did not consider left ventricular (LV) diastolic function and right ventricular (RV) function. We therefore examined the relation between NT-proBNP on LV and RV remodelling in an African hypertensive cohort.

**Methods:**

Subjects were subdivided into three groups after echocardiography: hypertensives without LVH (HT) (*n* = 83); hypertensives with LVH (HT+LVH) (*n* = 50); and those with hypertensive heart failure (HHF) (*n* = 77).

**Results:**

Subjects with HHF had significantly higher NT-proBNP levels compared to the HT+LVH group (*p* < 0.0002). NT-proBNP correlated positively with right atrial area, an indirect measure of RV function.

**Conclusions:**

NT-proBNP is proposed as a useful biomarker in differentiating hypertension with or without LVH from hypertensive heart failure in black hypertensive subjects.

## Abstract

Left ventricular hypertrophy (LVH) represents an important index of pre-clinical disease, and carries incremental prognostic value beyond that afforded by traditional coronary risk factors.^1^ In a large cohort of black persons, LVH proved to be an even more powerful predictor of mortality than coronary artery disease and left ventricular ejection fraction (LVEF).^2^ Hence early detection of LVH is very important in the management of the hypertensive patient.

Electrocardiography can be very useful in assessing LVH, especially in middle- and low-income countries, because it is relatively cheap, accessible and not much expertise is required to operate an electrocardiography machine. Electrocardiographic criteria for LVH are, however, not very sensitive, while the alternative more accurate method of echocardiography is uneconomical, especially in resource-limited countries.^3^ Besides requiring more expertise, the results may not be adequate in all patients, especially in those with obesity or pulmonary disease.^4^ This situation has led to research on the use of biomarkers such as NT-proBNP and BNP in the detection of the presence of LVH and monitoring its regression.^5^

B-type natriuretic peptide is a cardiac neurohormone secreted by myocardial cells located on both the atria and ventricles, mainly by LV myocardial cells in response to volume expansion and pressure overload.^6^,^7^ Plasma BNP and NT-proBNP levels are a useful marker of LVH in hypertension, and have also been found to rise progressively with increasing severity of hypertension, particularly when ventricular hypertrophy is present.^6^ Similarly, plasma BNP and NT-proBNP levels are useful to discriminate between patients with regard to cardiac remodelling and could be considered as a screening tool to select hypertensive patients eligible for transthoracic echocardiography.^5^ NT-proBNP is also a useful biomarker in differentiating hypertensive subjects with LVH from those with heart failure.^8^,^9^

Most of the current knowledge and published data on the use of plasma NT-proBNP in hypertensive LVH and hypertensive heart failure (HHF) are based on studies in Europe and the United States of America, with a dearth of data in black Africans in whom the burden of hypertension and hypertensive heart disease is very high.^10^,^11^ For example, the THESUS study, which studied 1 006 acute heart-failure subjects in nine sub-Saharan African countries, inclusive of Nigeria, showed that hypertension was the commonest cause of heart failure, accounting for heart failure in 45.4% of cases.^12^ In addition, most previous studies on this subject never considered LV diastolic function or RV function, both of which are reported to be prognostic markers in hypertensive heart failure.^13^,^14^ We therefore decided to examine the relationship between circulating NT-proBNP and left and right ventricular remodelling in a black African hypertensive cohort.

## Methods

This prospective cohort study was approved by the University of Abuja Teaching Hospital’s ethical clearance committee and is in compliance with the Helsinki declaration. The minimum age for participation in the study was 18 years but there was no upper age limit. Recruitment for the present study was initiated in December 2011 and data were obtained until August 2012.

Of the 220 patients with hypertension with or without heart failure enrolled for the study, 10, representing 4.5% of the total enrolment, were excluded because they were diabetic, had regional wall motion abnormality on transthoracic echocardiography, had serum creatinine greater than 170 μmol/l or acute myocardial infarction. Therefore, 210 subjects were studied, of whom 133 were subjects with a new referral for hypertension to the Cardiology Unit, Department of Medicine, University of Abuja Teaching Hospital, and 77 were subjects with hypertensive heart failure, presenting consecutively to the same unit.

Hypertension was defined according the JNC VII guidelines,^15^ while heart failure was diagnosed according to the guidelines of the European Society of Cardiology.^16^ The functional status of the HF subjects was according to the guidelines of New York Heart Association functional classification.^17^ All subjects gave written informed consent to participate in the study.

Each subject had fasting blood sugar level, fasting lipid profile, electrolyte, urea and creatinine levels, and full blood count assessed. Each subject also had blood collected, processed and plasma stored at –80°C until assayed for NT-proBNP. Subjects also had a transthoracic echocardiography performed on the same day that the sample was collected for NT-proBNP assay, the samples being analysed at the Hatter Institute, University of Cape Town.

All the subjects completed a standard questionnaire. Due to the multiplicity of languages in Nigeria, the questionnaire was not translated into any of the local languages. The majority of the subjects were reasonably proficient in the English language. Where there was a need for interpretation, both medical and paramedical staff of the Cardiology Unit of the Department of Medicine of University of Abuja Teaching Hospital assisted.

The questionnaire requested specific answers to date of birth, gender, occupation, background diagnosis of hypertension, background diagnosis of diabetes mellitus, history of angina pains, history of alcohol consumption and history of smoking habits. Details of anthropometric measurements, conventional blood measurements and assays for NT-proBNP have been reported in our previous publication.^18^

Echocardiography was performed using a commercially available ultrasound system (Vivid E). Subjects were examined in the left lateral decubitus position using standard parasternal, shortaxis and apical views. Studies were performed by an experienced echocardiographer according to the recommendations of the American Society of Echocardiography^19^

In our echocardiography laboratory, the intra-observer concordance correlation coefficient among the three cardiologists involved in the study ranged from 0.76–0.93, while that of the inter-observer concordance ranged from 0.82–0.95. Measurements were averaged over three cardiac cycles. The left and right atrial areas were measured at end-ventricular systole when the atrial chambers were at their greatest dimension, and with the bases of both atria at their greatest dimensions. Other details of our echocardiography measurements have been reported in our previous publication.^18^

## Statistical analysis

SPSS software version 16.0 (SPSS Inc, Chicago, IL) was used for statistical analysis. Continuous variables were expressed as mean ± SD. Comparison of demographic, clinical, laboratory and echocardiographic parameters among the three groups was performed by ANOVA test of variance. Correlation coefficients were calculated by linear regression analysis with serum NT-proBNP log-transformed to establish normality, and correlations between serum NT-proBNP and continuous demographic, clinical, laboratory and echocardiographic data were evaluated with Spearman’s regression.

Multivariate linear regression analyses were performed with log-transformed NT-proBNP concentrations as dependent variable, with the inclusion of demographic, clinical, laboratory and echocardiographic parameters. A two-tailed *p*-value < 0.05 was considered significant

## Results

Table 1 shows the demographic, clinical and laboratory characteristics of the subjects studied. Subjects with hypertensive HF had the lowest weight of the three study groups, with a body mass index of 25.4 ± 4.5 kg/m^2^ as against 27.6 ± 6.6 kg/m^2^ for subjects with hypertension with or without LVH (*p* = 0.03). Hypertensive subjects with LVH had the highest levels of mean arterial pressure and pulse pressure, while subjects with hypertensive HF had the lowest levels.

**Table 1:** Clinical profile of the subjects

*Parameters*	*All (n = 77)*	*Male (n = 54)*	*Female (n = 23)*	p*-value*
Age, years	53.8 ± 13.2	53.8 ± 15.8	51.7 ± 13.6	0.56
Smoking habits, *n* (%)	24 (13.1)	22 (18.6)	2 (2.5)	< 0.001
Body mass index, kg/m^2^	24.30 ± 7.0	24.2 ± 7.6	24.5 ± 5.9	0.86
Palpitations, *n* (%)	40 (51.9)	24 (44.4)	16 (69.6)	0.002
Peripheral oedema, *n* (%)	49 (63.2)	35 (64.8)	14 (60.8)	NS
NYHA class				
II, *n* (%)	14 (18.2)	10 (18.5)	4 (17.5)	
III, *n* (%)	49 (63.6)	35 (64.8)	14 (60.8)	
IV, *n* (%)	14 (18.2)	9 (16.7)	5 (21.7)	
SBP, mmHg	149.1 ± 23.8	149.9 ± 23.8	147.7 ± 23.9	0.55
DBP, mmHg	98.1 ± 13.9	98.2 ± 13.9	97.9 ± 13.9	0.92
PP, mmHg	55.8 ± 16.2	56.4 ± 16.8	54.7 ± 15.0	0.52
MAP, mmHg	101.3 ± 16.4	101.2 ± 17.2	101.5 ± 15.0	0.89
FBS, mmol/l	5.3 ± 2.2	5.2 ± 2.0	5.4 ± 2.4	0.58
Total cholesterol, mmol/l	4.2 ± 1.2	4.1 ± 0.2	4.3 ± 1.2	0.22
LDL cholesterol, mmol/l	2.7 ± 0.9	2.6 ± 1.0	2.8 ± 1.0	0.14
HDL cholesterol, mmol/l	1.1 ± 0.4	1.1 ± 0.4	1.1 ± 0.3	0.63
Estimated GFR, ml/min/1.73 m^2^	101.5 ± 38.8	111.6 ± 41.4	78.3 ± 17.0	< 0.0001
NT-proBNP, pg/ml	501.7 ± 199.8	513.0 ± 208.5	478.7 ± 184.7	0.58
Serum ST2, ng/ml	112.9 ± 78.7	100.1 ± 60.4	134.4 ± 98.3	0.26

SBP = systolic blood pressure, DBP = diastolic blood pressure, PP = pulse pressure, MAP = mean arterial pressure, FBS = fasting blood sugar, LDL = low-density lipoprotein, HDL = high-density lipoprotein, GFR = glomerular filtration rate.

There was no significant difference among the study populations in the levels of fasting blood sugar, fasting lipid profile, urea, creatinine, haemoglobin concentration and white blood cell count. There was also no significant difference in the NT-proBNP levels between the hypertensive subjects without and those with LVH.

**Fig. 1.**
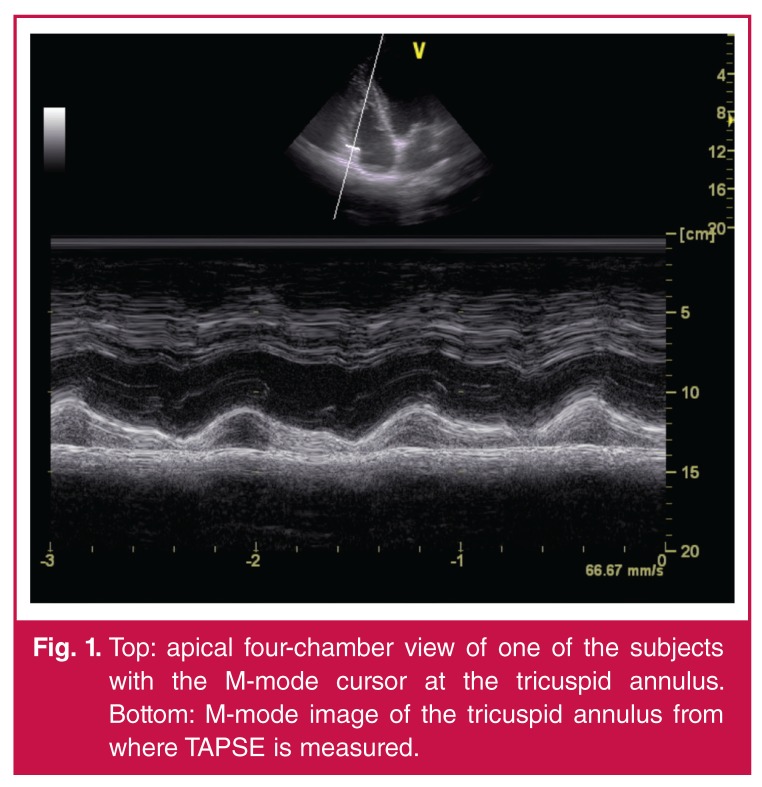
Top: apical four-chamber view of one of the subjects with the M-mode cursor at the tricuspid annulus. Bottom: M-mode image of the tricuspid annulus from where TAPSE is measured.

Fig. 1 shows the different concentrations of plasma NT-proBNP in the hypertensive cohort. Subjects with hypertensive HF had significantly higher NT-proBNP levels when compared with other hypertensive subjects, whether with or without LVH (*p* < 0.001).

Table 2 shows the echocardiographic characteristics of all the subjects studied. Hypertensive subjects with LVH had significantly higher interventricular and left ventricular posterior wall hypertrophy when compared with hypertensive subjects without LVH (*p* < 0.001 and 0.001, respectively), and when compared with subjects with hypertensive HF (*p* < 0.001). Hypertensive subjects with LVH also had higher LV mass and LV mass index when compared with hypertensive subjects without LVH and HF (*p* < 0.001). They had a smaller LV mass, whether indexed or not, when compared with hypertensive HF subjects (*p* < 0.001).

**Table 2:** Echocardiographic profile of the subjects

*Parameters*	*All (n = 77)*	*Male (n = 54)*	*Female (n = 22)*	*p-value*
RVD, cm	3.4 ± 0.6	3.5 ± 0.6	3.2 ± 0.5	0.22
Left atrial diameter, cm	4.6 ± 0.9	4.6 ± 0.9	4.5 ± 0.8	0.17
IVSDd, cm	1.1 ± 0.3	1.1 ± 0.2	1.0 ± 0.3	0.03
PWDd, cm	1.1 ± 0.2	1.2 ± 0.2	1.1 ± 0.2	0.07
EDD, cm	5.8 ± 1.1	5.9 ± 1.1	5.5 ± 1.1	0.04
ESD, cm	4.7 ± 1.3	4.9 ± 1.2	4.5 ± 1.3	0.07
LAA, cm^2^	24.5 ± 7.0	24.5 ± 6.7	24.4 ± 7.5	0.95
RAA, cm^2^	22.3 ± 8.1	22.6 ± 8.0	21.7 ± 8.5	0.50
LVM/height^2.7^	108.3 ± 46.3	117.5 ± 35.4	112.5 ± 42.3	0.65
LVEF, %	35.2 ± 17.5	34.4 ± 16.8	36.6 ± 18.7	0.58
ME, m/s	0.78 ± 0.3	0.76 ± 0.30	0.81 ± 0.30	0.43
MA, m/s	MA, m/s	0.49 ± 0.2	0.49 ± 0.1	0.25
ME/MA	2.2 ± 1.3	2.0 ± 1.2	2.2 ± 1.4	0.96
DT, ms	143.2 ± 80.6	143.7 ± 85.1	142.2 ± 72.2	0.22
TAPSE, mm	16.2 ± 5.1	16.6 ± 5.4	15.5 ± 4.5	0.16
TAPSE < 15 mm (%)	33 (42.9)	54 (40.7)	10 (41.7)	0.18
RVSP, mmHg	31.4 ± 10.5	31.4 ± 10.4	31.3 ± 10.5	0.97

RVD = right ventricular diameter in diastole, IVSDd = interventricular septal diameter in diastole, PWDd = posterior wall diameter in diastole, EDD = end-diastolic diameter, ESD = end-systolic diameter, LAA = left atrial area, RAA = right atrial area, LVM = left ventricular mass, LVEF = left ventricular ejection fraction, ME = early mitral inflow, MA = late mitral inflow, DT = deceleration time, TAPSE = tricuspid annular plane systolic excursion, RVSP = right ventricular systolic pressure.

Hypertensive subjects without LVH and left ventricular HF had the highest LV ejection fraction (*p* < 0.02) when compared with hypertensive subjects with LVH, and when compared with subjects with HF (*p* < 0.001). Apart from the right atrial area, hypertensive HF subjects had significantly higher chamber diameters. They also had the highest mitral E/A ratio and the lowest tricuspid annular plane systolic excursion value.

Pearson correlation analysis of clinical and echocardiographic variables with log-transformed NT-proBNP in the study population is shown in Table 3. NT-proBNP was significantly associated with left ventricular ejection fraction (*p* = 0.01) but not with tricuspid annular pulmonary systolic excursion (TAPSE). It was also significantly correlated with age (*p* < 0.04), pulse pressure and mean arterial pressure (*p* = 0.002 and *p* = 0.002, respectively), systolic blood pressure (*p* = 0.007), serum creatinine level (*p* = 0.038) and right atrial area (*p* < 0.0001). There was no significant correlation between NT-proBNP and body mass index, right ventricular diameter in diastole, interventricular septal wall thickness in diastole, posterior wall diameter in diastole, left atrial area, LV mass index, transmitral E/A ratio, deceleration time and TAPSE.

**Table 3:** Clinical and echocardiographic correlates of NT-pro BNP

*Parameters*	*Coefficient of association *(r)	p*-value*
Age (years)	0.17	0.04*
BMI	–0.07	0.40
Pulse pressure	0.26	0.002*
Mean arterial pressure	0.26	0.002*
IVSDd	0.17	0.05
PWDd	0.08	0.36
LVIDd	0.16	0.05
LVIDs	0.21	0.01*
RVD	0.09	0.31
LAA	0.02	0.80
RAA	0.20	0.04*
LVM/height^2.7^	0.09	0.30
LVEF	–0.21	0.01*
ME	0.02	0.79
MA	0.12	0.15
Mitral E/A ratio	0.08	0.35
Deceleration time	0.14	0.09
TAPSE	–0.23	0.15

IVSDd = interventricular septal diameter in diastole, PWDd = posterior wall diameter in diastole, LVIDd = left ventricular internal diameter in diastole, LVIDs = left ventricular internal diameter in systole, RVD = right ventricular diameter in diastole, LAA = left atrial area, RAA = right atrial area, LVM = left ventricular mass, EF = ejection fraction, ME = early mitral inflow, MA = atrial or late mitral inflow, TAPSE = tricuspid annular plane systolic excursion. *Significant at *p* < 0.05.

In multivariate linear regression analysis (Table 4), independent predictors of NT-proBNP in the study population included LV ejection fraction (*t* = 2.11; *p* = 0.037), right atrial area (*t* = 1.99; *p* = 0.048) and LV internal diameter in systole (*t* = 2.21; *p* = 0.029).

**Table 4:** Univariate analysis with right ventricular systolic pressure and cardiac biomarkers

*Parameter*	*Pearson correlation*	*p-value*
Serum ST2	0.75	< 0.0001
NT-proBNP	0.54	< 0.0001

## Discussion

This study has shown that NT-proBNP differentiates hypertensive LVH from hypertensive HF not only in Caucasians,^9^ but also in black African hypertensive subjects. We found no significant difference in the concentrations of NT-proBNP between hypertensive subjects with LVH and those without LVH, which is in keeping with previous findings.^5^,^24^,^25^ NT-proBNP concentrations were not correlated with LV mass index, interventricular septal wall thickness or posterior wall thickness in diastole, which is similar to other findings.^9^ This lack of correlation between NT-proBNP and LV mass index might explain why NT-proBNP is not a good marker for differentiating hypertensive LVH from hypertension without LVH and HF.

NT-proBNP correlated with both mean arterial pressure and pulse pressure. Age and plasma creatinine levels were found to correlate with NT-proBNP concentration in our study, in keeping with previous reports that NT-proBNP rises with increasing age,^26^,^27^ and worsening renal status.^28^

Similar to previous findings, we showed no correlation with deceleration time and trans-mitral E/A ratio, which are indices of left ventricular function. Richard *et al.*,^31^ however, found a relationship between LV diastolic function and plasma BNP levels using newer diastolic indexes measured from tissue Doppler imaging and colour M-mode that allow more accurate characterisation of myocardial relaxation and left ventricular filling.

Unlike some previous studies, our study did not only assess remodelling of the left-sided chambers and LV systolic function, but also remodelling of the right heart chambers, LV diastolic function and right ventricular systolic function.

Even though there was no significant correlation between the concentration of NT-proBNP and TAPSE, the right atrial area, which is a measure of remodelling of the right cardiac chamber and an indirect measure of right ventricular function, correlated significantly with NT-proBNP. This suggests right cardiac chamber remodelling had some effect on the concentration of plasma NT-proBNP in our hypertensive cohort. Correlation between BNP and right atrial size has been previously described.^32^,^33^

Hypertensive subjects with LVH had significantly worse LV systolic function compared to subjects without LVH (*p* < 0.02), which may support the fact that hypertensive subjects with LVH have worse cardiovascular profile compared to those without hypertrophy.^34^

Our subjects with hypertensive HF were much younger, with a mean age of 53.0 ± 11.9 years compared to the developed countries where HF is a disease of the elderly, with an average age of 76 years.^35^,^36^ Hypertensive HF presenting in a relatively young cohort in this Nigerian population is a reflection of the presentation of the complications of hypertension at an early stage.

Long distance and often lack of funding to cover the travel fare are important aspects of late presentation to healthcare.^37^ This presentation of hypertensive HF at a relatively early age has the potential to undermine national productivity as a consequence of the number of active life years lost by the most active workforce of the population.

## Conclusion

This study has shown that NT-proBNP is a good marker in differentiating hypertensive HF from hypertension with or without LVH. Our finding supports the need to introduce NT-proBNP point-of-care machines^39^ in our cardiology practices in sub-Saharan Africa. Currently, the use of point-of-care tests in resource-limited settings such as ours has focused mainly on infectious diseases that need prompt diagnosis and treatment, such as HIV infection, tuberculosis and malaria,^40^ and diabetes care.^41^

Therefore the need for the introduction of point-of-care NT-proBNP assays for early diagnosis while awaiting echocardiography in our cardiology practice cannot be overemphasised. For such a point-of-care test to be very effective in the sub-continent, there is a need to further reduce the cost of these devices compared with what is obtainable in Europe and the United States.
